# Evaluation of Aspirin and Clopidogrel resistance in patients with Acute Coronary Syndrome by using Adenosine Diposphate Test and Aspirin Test 

**DOI:** 10.12669/pjms.291.2820

**Published:** 2013

**Authors:** Ibrahim O, Oteh M, A Syukur A, Che Hassan HH, S Fadilah W, MM Rahman

**Affiliations:** 1 Ibrahim O, Department of Medicine, Faculty of Medicine, University Kebangsaan Malaysia Medical Centre, Cheras 56000, Kula Lumpur, alaysia.; 2 Oteh M, Consultant Cardiologist, Unit Cardiology, Department of Medicine, Faculty of Medicine, University Kebangsaan Malaysia Medical Centre, Cheras 56000, Kula Lumpur, alaysia.; 3 A Syukur A, Department of Medicine, Faculty of Medicine, University Kebangsaan Malaysia Medical Centre, Cheras 56000, Kula Lumpur, alaysia.; 4 Che Hassan HH, Department of Medicine, Faculty of Medicine, University Kebangsaan Malaysia Medical Centre, Cheras 56000, Kula Lumpur, alaysia.; 5 S Fadilah W, Consultant Haematologist and Head, Department of Medicine, Faculty of Medicine, University Kebangsaan Malaysia Medical Centre, Cheras 56000, Kula Lumpur, alaysia.; 6 MM Rahman, Professor, Department of Medical Microbiology & Immunology, Faculty of Medicine, University Kebangsaan Malaysia Medical Centre, Cheras 56000, Kula Lumpur, alaysia.

**Keywords:** Anti-platelet therapy, Acute coronary syndromes, Aspirin, Clopidogrel, Hyperlipidaemia

## Abstract

***Objectives:*** To evaluate Aspirin and Clopidogrel resistance/non-responders in patients with acute coronary syndrome (ACS) by using adenosine diposphate and aspirin tests.

***Methodology:*** In the study patients with ACS loaded with 300 mg of clopidogrel and 300 mg aspirin and patients on stable daily dose of 75 mg of clopidogrel (more than 3 days) underwent PCI. Response to clopidogrel and Aspirin was assessed by Adenosine Diphosphate (ADP) Test (20 µmol/L) and Aspirin Test (Acetyl Acid) (ASP) 20 µmol/L, respectively, using the Multiplate Platelet Function Analyzer (Dynabyte Medical, Munich, Germany).

***Results: ***Sixty four patients were included in this study out of which 57 were with ACS and 7 scheduled for percutaneous coronary intervention (PCI) electively. The proportion of Aspirin good responders and adequate responders were 76.56% and 18.75%, respectively while adequate response and good response to Clopidogrel accounted for 29.7 and 48.4%, respectively Hyperlipidaemia was only co-morbidity associated with higher AUC ADP value (p: 0.046). Hypertriglyceridaemia and serum calcium were weakly correlated with higher AUC ADP serum calcium r=0.08, triglyceride r=0.12. Patients admitted for scheduled PCI and on stable dose of 75mg clopidogrel exhibited lower AUC ADP value as compared to those admitted with acute coronary syndrome given loading dose of 300mg of Clopidogrel. Post loading dose measurement of anti-platelet therapy among ACS patients using the Multiplate Platelet Function Analyzer showed comparable results with other methods.

***Conclusions*** **:** As determined by Multiplate Platelet Function Analyzer, Aspirin resistance/non-responders in this study in acute coronary syndrome patients accounted for 4.69% while Non-responders in Clopidogrel was 21.9%.

## Introduction

 Anti-platelet therapy remains most important and effective management in prevention of important clinical complications of atherothrombosis namely acute coronary events, cerebral vascular accidents and all other thrombotic events. Platelet activation and aggregation plays a pivotal role in such cases. The Anti-platelet trials documented the effect of aspirin on more than 100,000 aspirin-treated patient and controls, highlighting 25% reduction of death, myocardial infarction and stroke in high-risk vascular patients, 48% reduction in vascular graft and arterial occlusion, 67% reduction of pulmonary embolism and 23% reduction of deep vein thrombosis.^1^ A study conducted to compare the activity of clopidogrel and aspirin in ischaemic events showed the superiority of clopidogrel in preventing cardiovascular events.^2^ Several trials has demonstrated the reduction of cardiovascular risks by dual anti-platelet therapy with the combination of aspirin and clopidogrel, a theinopyridine that causes irreversible inhibition of the platelet ADP receptor P2Ysub_12_.^3^^-^^5^ Platelet aggregometry is most often used to assess and measure platelet aggregation. However, laboratory definitions of non-responders have varied according to the platelet function tests used, and no study has prospectively validated conventional platelet aggregometry as an independent predictor of subsequent serious vascular events.^6^

 In this study, platelet function analysis was performed using the Multiplate® platelet analyser (Dynabyte, Munich, Germany. It has been used to study the effects of aspirin^7^^,^^8^, non opiod analgesics^9^, clopidogrel^10^^,^^11^, anticoagulants^12^, antifibrinolytics^13^, colloids^14^ and temperature^15^, on platelet aggregation. Multiplate® platelet analyser analyzes whole blood impedance aggregometer. This aggregometry is based on the principle that blood platelets are non-thrombogenic in their resting state but expose receptors on their surface when they get activated which allow them to attach on vascular injuries and artificial surfaces. 

 The aim of the study was mainly to determine the incidence of aspirin and clopidogrel non-responders among local population and the predictors and consequences of platelet non-inhibition in patient going for percutaneous coronary intervention measured by the Multiplate® platelet analyser (Dynabyte, Munich, Germany).

## Methodology

***Patient population: ***This cross-sectional prevalence study was conducted at University Kebangsaan Malaysia Medical Centre (UKMMC) between February to April 2009. The study was performed according to the Declaration of Helsinki upon approval by the ethics committee of UKMMC (FF**-**094-2009).

 All adult patients with Ischaemic Heart Disease (IHD) presented either with acute coronary syndromes (ACS), ST MI, non ST elevation myocardial infarction, unstable Angina or electively admitted for percutaneous coronary intervention (PCI) were included in the study. Target sample size was 136. 

***Patient assessment and blood sample collection: ***Clinical history was taken and investigations were performed on the patients following the inclusion criteria after taking their informed consent. The investigations of full blood count (FBC), renal profile (RP), liver function test (LFT), fasting blood sugar (FBS), total Cholesterol, Low density Lipoprotein (LDL), High density Lipoprotein (HDL), Triglycerides (TGs), Serum calcium, cardiac enzymes, Troponin T after collecting blood from the patients. Chest x-ray and echocardiogram were also done immediately after admission. The patients who consented for PCI were pre-loaded with clopidogrel 300mg at least 24 hours prior to procedure, followed by maintenance dose of 75 mg daily. Few patients with stable dose of 75mg of clopidogrel were also included in this study and these patients should have been on Clopidogrel at least three days. Patients with ACS were given 300 mg loading of aspirin and subsequently maintenance dose of 150 mg of daily. Whole blood was collected using heparin as anticoagulants 24 hours after the clopidogrel loading dose or 72 hours later if not given clopidogrel loading dose, 5 ml each in a Lithium Heparin bottle (non-gel) using the Multiplate Platelet Function Analyzer (Dynabyte Medical, Munich, Germany) 2006, at activator concentration pf 20µl, the blood samples were analyzed. 

***Method of performing multi-plate test: ***The test cells were put into the measuring position attaching the sensor cable, pipette 300 µL of saline (pre-warmed at body temperature) then 300 µL of whole blood sample (hirudinised) into each of the test cells. Later on these were allowed 3 minutes for warming and equilibrated before adding the activator and waited for the results after 6 to 10 minutes. Finally the results were printed with graph produced by each test showed aggregation in AU unit, velocity and Area under the curve.

***Definition of aspirin and clopidogrel non responders: ***Aspirin and clopidogrel non-responders were defined as reduction of more or equal to 30% of normal reference range given by machine manufacturer, Multi-plate Analyzer (Table-I). This figure was derived from few studies which used the range of less than 20 to 30% reduction between the normal subjects or pre-treatment group (baseline) and post-treatment were non responders. Hence, levels above the estimated post-treatment range were considered non responders.

**Table-I:** Multi-plate reference range for AUC ASP and AUC ADP Test**

*Test: Activator*	*ADP Test ADP*	*ASP Test Arachidonic Acid*
Volume	20 µl	20 µl
Final Concentration	6.4 µM	0.5 µM
Parameter	AUC	AUC
Reference Range	607-963	505-1086
Estimated Post- Treatment Range	424-674	353-760
	< 424 = good responder	<353 = good responder
	424-674 = adequate responder	353-760 = adequate responder
	>674 = non responders	>760 = non responders

** Dynabyte medical-Cornelius Str.28-80469 Munich, Germany 2006.

***Statistical analysis: ***Data were analyzed using the SPSS version 15.0 statistical package. Quantitative and qualitative demographic characteristics were summarized and data were tabulated and tested for normality with Shapiro-Wilk test because the sample was below 100. All statistical tests were carried out at a significant level of p value < 0.05. Data were expressed as mean +/- standard deviation (SD), median (95%CI) and inter-quartile range (IQR) or proportion or percentage for all data.

**Table-II:** Demographic and Clinical Characteristics of the patients.

*Characteristic*	*N=64*
Mean age-Years	58.56 ± 11.84 ^a^
Male sex- no (%)	52(81.25)
Weight (kg)	70.00(16.00)^b^
Reason for admission-no (%)	
-STEMI	13(20.31)
-NSTEMI	21(32.81)
-Unstable angina	23(35.94)
-Elective PCI	7(10.94)
Fasting lipid profile	
-LDL (mmol/l)	3.40 (1.39)^a^
-HDL (mmol/l)	0.98(0.49)^b^
-Triglyceride (TG) (mmol/l)	1.67(1.27)^b^
-Total cholesterol	4.93(1.76)^b^
Serum calcium	2.24(0.16)^b^
Echocardiogram (Ejection Fraction)	52.29 ± 12.61^a^
HbA1c	7.1(3.10)^b^
Random Blood Sugar (RBS)	8.6(6.8)^b^

a=mean + SD (standard deviation),

b = Median (IQR; interquartile range)

## Results

***Patients’ demography: ***Sixty four patients were recruited from the cardiac care unit and medical wards with the following demographic data and clinical characteristics (Table-II). The mean AUC ADP for the study patients was 482.61 ± (SD 231.82). The mean aggregation for the ADP Test was 88.67 ± (SD40.80) and the velocity (AU/min) was 10.20 (IQR 6.88). For ASP Test, the AUC ASP was 237 (IQR 189.5) with median aggregation and velocity were calculated at 43.85 + (IQR 34.33) and 6.45 (IQR 4.43) respectively (Table-III). The proportion of both aspirin and clopidogrel were calculated by the defined estimated range by the Multi-plate Analyzer into three groups. The proportion of aspirin good responders and adequate responders were 76.56% and 18.75%, respectively. Aspirin non responder group only accounted for 4.69% of the patients and in the clopidogrel group 14 patients were deemed non responsive to the treatment which totaling 21.9% of overall patients. Others were either manifested as adequate response and good response to clopidogrel which accounted for 29.7 and 48.4%, respectively (Table-IV).

**Table-III:** Results for Median Aggregation Test Both Aspirin (ASP) Test and Mean Adenosine Diphosphate (ADP) Test.

	*Result*
ASP TEST	
Aggregation (AU)	43.85 ± (IQR 34.33)
Velocity (AU/min)	6.45 ± (IQR 4.43)
Area Under The Curve (AUC)	237 ± (IQR 189.5)
(AU^x^min)	
ADP TEST	
Aggregation (AU)	88.67 ± (SD 40.80)
Velocity (AU/min)	10.20 ± (IQR 6.88)
Area Under The Curve (AUC)	482.61 ± (SD 231.82)
(AU^x^min)	

**Table-IV:** Proportion of Aspirin and Clopidogrel Responders by Multiplate test.

	*Aspirin*	*Clopidogrel*
Good Responder	49 (76.6%)	31 (48.4%)
Adequate Responder	12 (18.7%)	19 (29.7%)
Non Responder	3 (4.7%)	14 (21.9%)

 Hyperlipidaemia has significantly contributed to the higher level of AUC ADP in this study. Patients who have this medical problem is likely to be in non responder group for clopidogrel (p value = 0.046). However, it was not associated in predicting aspirin non responder (p value = 0.632). Diabetes which is generally accepted as risk equivalent to coronary heart disease showed no significant value in halting response towards both anti-platelets (p value : 0.188 and 0.310) Other co morbidities studied such as hypertension, established coronary heart disease, smoking, alcohol consumption and renal disease were not influencing the response towards treatment with both anti platelets. In correlating AUC ADP Test with the quantitative variables using binary logistic regression, serum calcium and serum triglyceride level were positively correlated in weak manner with the test done, the p values for the 2 variables were 0.049 and 0.005 with r value of 0.08 and 0.12, respectively. However, there was no correlation between age, sex, weight, height, Body Mass Index (BMI) and ejection fraction on the echocardiogram.

 One way ANOVA was used in assessing relationship between reasons of admission and the AUC ADP value showed that the stable clopidogrel dose patients (n=7) who came for elective percutaneous coronary intervention had lower AUC ADP value compared to NSTEMI group (p value = 0.034) hence lower risk of non responding to treatment.

 The use of concurrent drugs to treat medical illness showed no statistical significance in influencing the aspirin and clopidogrel efficacy. Independent T Test for the group of proton pump inhibitor, calcium channel blocker and statins users has no significant effect on the tests

## Discussion

 This was cross-sectional prevalence study looking at the group of coronary heart disease patients presenting with acute coronary syndromes or electively admitted for PCI. We wanted to determine the proportion of patients who were termed anti-platelet non responders or resistance, male outnumbered female since the risk of getting ACS among other previous studies showed more men carry risk factors and co-morbidities as compared to female counterpart. Non ST Elevation Myocardial Infarction was observed to be the main hospital diagnosis admission for ACS. We included 7 patients of elective cases on stable dose (more than 3 days) of clopidogrel 75 mg daily to compare the platelet aggregation as compared to patients who were admitted with ACS and loaded with 300 mg of clopidogrel. In this situation, the understanding of clopidogrel effects after 72 hours on stable dose 75 mg daily, 24 hours after loading of 300 mg of clopidogrel determine our blood analysis sample collection to observe maximal inhibition of platelet aggregation. We did not practice 600 mg of loading dose of clopidogrel since few studies showed that the effects of different dose did not outwit each other and possible conferred more adverse events.^16^ AUC ADP and AUC ASP are quantitative measurements of platelet aggregation which could be measured by various type of platelet aggregometry.

 Multiple studies previously detected aspirin and clopidogrel via the classical Light Tranmission Aggregometry (LTA).^17^ Multiplate Analyzer used in this study worked in whole blood through an impedance technique. A study by Corina et al^11^ compared the two devices and techniques concludes that the results achieved with the bedside Multiplate assays were not different from those obtained with classical aggregometry for detecting the effects of aspirin and clopidogrel. There is variability in prevalence of aspirin resistance and non-responder world-wide that may suggest gene polymorphism in some group population.^18^ Asian study by Lee et al^19^ performed on patients going for percutaneous coronary intervention observed 19% aspirin resistant. In this study we were able to show that the proportion of aspirin non responders was significantly lower in our population 4.69% while that of clopidogrel follows most of the studies prevalence was 21.9% of overall patients.

 Patients with higher triglyceride level and being diagnosed as hyperlipidaemia as we can see from the study were positively correlated with higher AUC ADP value which could be part of metabolic syndrome. Serebruany et al^20^ showed closure time was shorter in patients with metabolic syndrome indicating platelet inhibition under high shear condition and increased expression of PAC-1 that strongly suggested activation of platelet glycoprotein IIb/IIIa receptor.

 The proportion of known diabetes in our study justifies diabetes as a major risk factor. However, analysis in our data showed no statistical difference due to the patients enrolled in this study were in better control of diabetes if we take into consideration the median of HB1Ac of 7.1% and Random Blood Sugar of 8.6.^21^ Serum calcium in this study was weak predictive value (p: 0.049, r = 0.08) as it was correlated well with higher AUC ADP value. This is against a report by Japanese investigators that serum calcium was inversely correlated with the platelet aggregation but that study focussed on high salt intake hypertensive patient which oral calcium was thought to attenuate intracellular sodium retention.^22^

 The present study demonstrated that there was no significant relation in drug interaction with concomitant use in managing the most of ACS patients (54/64) with anti-platelet particularly clopidogrel and proton pump inhibitor (PPI). Michael Ho et al^23^ reported more adverse outcome with concomitant use of PPI and clopidogrel after hospital discharge for acute coronary syndromes. One interesting finding we found from the study was that the stable dose patients who were on 75 mg of clopidogrel have significantly better AUC ADP as compared to NSTEMI group after receiving loading therapy. In this regard, Serebruany et al^24^ reported that patient with stable dose proved consistent platelet inhibition.

 In this study, platelet function analysis was performed using the Multiplate® platelet analyser (Dynabyte, Munich, Germany), whole blood impedance aggregometer which was proved to be sensitive for all three classes of commonly applied platelet function inhibitors: COX inhibitors (aspirin^®^), ADP receptor antagonists (Clopidogrel, Prasugrel, Cangrelor) and IIb/IIIa antagonists (Reopro^®^, tirofiban, Integrilin^®^). Similar results were also observed by Sibbing et. al.^10^ Further study in this regard may be carried out with large sample size to provide comprehensive results on anti-platelet therapy and non responding patients.

## References

[1] Anti-thrombotic Trialist’s Collaboration (ATTC) (2002). Collaborative meta-analysis of randomised trials of anti-platelet therapy for prevention of death, myocardial infarction, and stroke in high risk patients. BMJ.

[2] Yusuf S, Zhao F, Mehta SR, Chrolavicius S, Tognoni G, Fox KK (2001). Effects of clopidogrel in addition to aspirin in 45, 852 patients with acute coronary syndromes without ST- segment elevation. N Engl J Med.

[3] Chen ZM, Jiang LX, Chen YP, Xie JX, Pan HC, Peto R (2005). Addition of clopidogrel to aspirin in 45,852 patients with acute myocardial infarctions: randomised placebo-controlled trial. Lancet.

[4] Bhatt DL, Fox KA, Hacke W, Berger PB, Black HR, Boden WE (2006). Clopidogrel and aspirin versus aspirin alone for the prevention of atherothrombotic events. NEJM.

[5] Sabatine MS, Cannon CP, Gibson CM, López-Sendón JL, Montalescot G, Theroux P (2005). Addition of clopidogrel to aspirin and fibrinolytic therapy for myocardial infarction with ST-segment elevation. N Engl J Med.

[6] Gurbel PA, Bliden KP, DiChiara J, Newcomer J, Weng W, Neerchal NK (2007). Evaluation of dose-related effects of aspirin on platelet function. Results from the aspirin-induced platelet effect (ASPECT) study. Circulation.

[7] Von PapeKW, Dzijan-Horn M, Bohner J, Spannagl M, Weisser H, Calatzis A (2007). Control of aspirin effect in chronic cardiovascular patients using two whole blood platelet function assays PFA-100 and Multiplate. Hamostaseologie.

[8] Rahe-Meyer N, Winterhalter M, Hartmann J, Pattison A, Hecker H, Calatzis A (2008). An evaluation of cyclooxygenase-1 inhibition before coronary artery surgery: aggregometry versus patient self-reporting. Anesth Analg.

[9] Jambor C, Weber CF, Lau A, Spannagl M, Zwissler B (2009). Multiple electrode aggregometry for ex-vivo detection of the anti-platelet effect of non-opioid analgesic drugs. Thromb Haemost.

[10] Sibbing D, Braun S, Jawansky S, Vogt W, Mehilli J, Schömig A (2008). Assessment of ADP-induced platelet aggregation with light transmission aggregometry and multiple electrode platelet aggregometry before and after clopidogrel treatment. Thromb Haemost.

[11] Velik-Salchner C, Maier S, Innerhofer P, Streif W, Klingler A, Kolbitsch C (2008). Point-of-care whole blood impedance aggregometry versus classical light transmission aggregometry for detecting aspirin and clopidogrel: the results of a pilot study. Anesth Analg.

[12] Sibbing D, Busch G, Braun S, Jawansky S, Schomig A, Kastrati A (2008). Impact of bivalirudin or unfractionated heparin on platelet aggregation in patients pretreated with 600 mg clopidogrel undergoing elective percutaneous coronary intervention. Eur Heart J.

[13] Mengistu AM, Rohm KD, Boldt J, Mayer J, Suttner SW, Piper SN (2008). The influence of aprotinin and tranexamic acid on platelet function and postoperative blood loss in cardiac surgery. Anesth Analg.

[14] Boldt J, Wolf M, Mengistu A (2007). A new plasma-adapted hydroxyethylstarch preparation: in vitro coagulation studies using thrombelastography and whole blood aggregometry. Anesth Analg.

[15] Scharbert G, Kalb M, Marschalek C, Kozek-Langenecker SA (2006). The effects of test temperature and storage temperature on platelet aggregation: a whole blood in vitro study. Anesth Analg.

[16] Geisler T, Langer H, Wydymus M, Gohring K, Zurn C, Bigalke B (2006). Low response to clopidogrel is associated with cardiovascular outcome coronary stent implantion. Eur Heart J.

[17] Abaci A, Caliskan M, Bayram F, Yilmaz Y, Cetin M, Unal A (2006). A New definition of aspirin non-responsiveness by Platelet Function Analyzer-100TM and its predictors. Platelets.

[18] Angiolillo DJ, Fernandez-Ortiz A, Bernardo E, Ramírez C, Escaned J, Moreno R (2004). 807 C/T Polymorphism of the glycoprotein Ia gene and pharmacogenetic modulation of platelet response to dual anti-platelet treatment. Blood Coagul Fibrinolysis.

[19] Lee PY, Chen WH, Ng W, Cheng X, Kwok JY, Tse HF (2005). Low-dose aspirin increases aspirin resistance in patients with coronary artery disease. Am J Med.

[20] Serebruany VL, Malinin A, Ong S, Atar D (2008). Patients with metabolic syndrome exhibit higher platelet activity than those with conventional risk factors for vascular disease. J Thromb Thrombolysis.

[21] Watala C, Golanski J, Pluta J, Boncler M, Rozalski M, Luzak B (2004). Reduced sensitivity of platelets from type 2 diabetic patients to acetylsalicylic acid (aspirin)-its relationto metabolic control. Thromb Res.

[22] Saito K, Sano H, Kawahara J, Yokoyama M (1995). Calcium supplementation attenuates an enhanced platelet function in salt-loaded mildly hypertensive Patients. Hypetension.

[23] Ho PM, Maddox TM, Wang L, Fihn SD, Jesse RL, Peterson ED (2009). Risk of Adverse Outcomes Associated With Concomittant Use of Clopidogrel and Proton Pump Inhibitors Following Acute Coronary Sydrome. JAMA.

[24] Serebruany VL, Malinin A, Atar D, Hanley DF (2007). Consistent platelet inhibition during long-term maintenance-dose clopidogrel therapy among 359 compliant outpatients with documented vascular disease. Am Heart J.

